# Exploratory Analysis of Phenolic Profiles and Antioxidant Capacity in Selected Romanian Monofloral Honeys: Influence of Botanical Origin and Acquisition Source

**DOI:** 10.3390/antiox14101248

**Published:** 2025-10-17

**Authors:** Elena Daniela Bratosin, Delia Mirela Tit, Anamaria Lavinia Purza, Manuela Bianca Pasca, Gabriela S. Bungau, Ruxandra Cristina Marin, Andrei Flavius Radu, Daniela Gitea

**Affiliations:** 1 Doctoral School of Biological and Biomedical Sciences, University of Oradea, 410087 Oradea, Romania; bratosin.elenadaniela@student.uoradea.ro (E.D.B.); gbungau@uoradea.ro (G.S.B.); marin.ruxandracristina@student.uoradea.ro (R.C.M.); andreiflavius.radu@uoradea.ro (A.F.R.); 2 Department of Pharmacy, Faculty of Medicine and Pharmacy, University of Oradea, 410028 Oradea, Romania; bpasca@uoradea.ro (M.B.P.); dgitea@uoradea.ro (D.G.); 3 Department of Psycho-Neurosciences and Recovery, Faculty of Medicine and Pharmacy, University of Oradea, 410073 Oradea, Romania

**Keywords:** monofloral honey, botanical origin, phenolic profile, antioxidant capacity, HPLC-DAD-ESI^+^, Romania

## Abstract

This exploratory study assessed the influence of botanical origin and acquisition source on the phenolic profile and antioxidant properties of selected Romanian monofloral honeys. Eight samples were analyzed, representing five floral types: acacia, linden, rapeseed, lavender, and thyme. For acacia, linden, and rapeseed, both commercial and locally sourced honeys were included. Analytical techniques included total phenolic content (TPC, Folin–Ciocalteu), antioxidant assays (DPPH, ABTS, FRAP), color intensity (ABS_450_), and phenolic compound profiling via HPLC-DAD-ESI^+^. TPC ranged from 179.26 ± 23.57 to 586.67 ± 18.33 mg GAE/100 g, with thyme and linden honeys presenting the highest values. Seventeen phenolic compounds were tentatively identified; gallic acid was predominant in thyme honey (127 mg/100 g), and linden honey contained high levels of rutin (70 mg/100 g) and galangin-glucoside. Antioxidant capacity varied notably by floral origin, with thyme and linden outperforming acacia samples. Significant correlations were found between total phenolics and ABTS (r = 0.86), and between ABS_450_ and FRAP (r = 0.86). DPPH kinetics followed zero-order behavior (*R*^2^ > 0.98). Principal component analysis (PC1 + PC2 = 88%) enabled preliminary separation by botanical origin. While based on a limited sample set, findings support the relevance of combining chromatographic, kinetic, and multivariate tools for exploratory honey characterization.

## 1. Introduction

Honey is recognized as one of the most intricate natural substances, rich in bioactive compounds that underpin its nutritional relevance and biological activities. Derived from a diverse array botanical origin honey exhibits substantial compositional variability, leading to differences in antioxidant and related properties. Historically valued in human diets and traditional applications such as wound care and respiratory health [1], honey has more recently become the focus of scientific investigation. Advances in analytical chemistry have clarified its phytochemical profile, highlighting phenolic acids and flavonoids as key contributors to antioxidant [2], anti-inflammatory [3], antimicrobial [4] and wound-supportive [5] properties. Phenolic compounds, including phenolic acids and flavonoids, significantly contribute to the antioxidant capacity of honey, with the amount varying according to the botanical source of the honey. The total phenolic content of honey and its in vitro antioxidant activity has been found to be directly positively correlated in several studies. Darker-colored honeys, which are frequently identified by increased absorbance at 450 nm, generally have more phenolic content and more potent antioxidant properties than lighter honeys [6,7]. The color intensity of honey (absorbance at 450 nm minus 720 nm) has thus been used as an indicator of antioxidant pigment content (flavonoids, carotenoids, Maillard reaction products) and shows a clear relationship with polyphenol levels [8]. Monofloral honeys often carry distinctive phenolic fingerprints, and marker compounds have been reported for Manuka, Kanuka, and sage honeys [9,10,11]. Such compositional distinctions are linked to differences in antioxidant and antimicrobial properties [4,12,13].

Moreover, the total phenolic content (TPC) and total flavonoid content (TFC) of honey can vary not only with botanical source but also with geographical and environmental factors. Plant nectar composition is influenced by soil and climate, which in turn shape the phenolic content of the resulting honey. Thus, honeys of the same floral type may show different TPC/TFC values depending on their geographical origin. For instance, linden (*Tilia* spp.) or acacia *(Robinia pseudoacacia*) honeys collected in different climates or elevations can differ quantitatively in their phenolic profiles. In this sense, Chilean honeys have been shown to cluster together based on their phenolic and flavonoid composition, exhibiting profiles distinct from honeys produced in other regions, while samples from the same Chilean areas share similar compositional traits [14,15,16]. Romanian monofloral and polyfloral honeys have been shown to contain varying levels of TPC, depending on the floral origin. Studies have reported TPC values ranging from 14.5 to 75 mg GAE/100 g in various samples, with forest and polyfloral honeys usually showing higher levels compared to acacia honey. Specific phenolic acids, such as gallic, caffeic and p-coumaric acids, as well as flavonoids such as quercetin and pinocembrin, have been identified by HPLC in Romanian honeys. In particular, lime and lavender honeys showed TPC values up to 50–58 mg GAE/100 g, while dark honeys, such as manna honey, reached over 110 mg GAE/100 g [17].

Phenolic compounds are therefore not only central to honey’s antioxidant potential but also serve as biomarkers for botanical and geographical origin. Their levels and profiles underpin sensory attributes such as color and flavor and are increasingly used to differentiate honeys of local or regional provenance. From this perspective, detailed phenolic characterization can contribute both to nutritional evaluation and to authenticity control, supporting the development of quality labels such as protected designation of origin or protected geographical indication [18].

Given the growing interest in the health-promoting properties of honey, as well as the growing concerns regarding its authenticity and standardization, studies focused on the identification and quantification of individual phenolic compounds have become essential. However, increasing evidence shows that both the botanical origin and the source of purchase (direct from producers or from stores) play an essential role in its chemical composition and bioactive potential [19]. Such variability highlights the need for detailed characterization of monofloral honeys to better understand the links between composition and antioxidant functionality. In this frame, our research aims to provide a preliminary characterization of selected Romanian monofloral honeys by combining phenolic profiling with antioxidant evaluation. By focusing on a limited set of honeys from different botanical origins and supply sources (local producers and commercial retail), the work provides baseline data that may support future, more extensive surveys and contribute to the understanding and valorization of Romanian honeys in the context of functional food research.

## 2. Materials and Methods

### 2.1. Honey Samples

This study investigated five Romanian monofloral honey types, as follows: acacia *(Robinia pseudoacacia* L.), rapeseed (*Brassica napus* L.), linden (*Tilia* spp.), lavender (*Lavandula angustifolia* L.), and thyme (*Thymus* spp.), which had been previously characterized in terms of botanical origin and physicochemical properties [20]. Also, sampling methods and analyzed samples, floral authentication, and sourcing regions are the same as those already detailed in our prior publication [15]. In brief, honey was collected in 2022 from two sources: raw, unprocessed honey directly obtained from certified local producers (coded as “_P”) and commercially available honey from national retail chains (coded as “_C”) (Table 1). Due to limited availability on the market, lavender and thyme honeys were available only from producers. This reflects their more niche production and lower retail distribution in Romania.

The samples originated from several Romanian counties (i.e., Brăila, Buzău, Vaslui, and Sălaj) and were selected based on floral availability rather than geographical representativeness. All honey samples were stored in sealed glass containers under controlled laboratory conditions (dark, 20 ± 2 °C, relative humidity below 50%) and were analyzed without undergoing any further processing.

Given the intrinsic variability of honey composition, we acknowledge that the limited number of samples per honey type represents a constraint of the present work. Thus, the results should be regarded as preliminary and exploratory, offering baseline data that can inform and guide more extensive, statistically powered surveys in the future.

### 2.2. Chemicals

Table 2 summarizes the used reagents. The ultrapure water was obtained using the Direct-Q UV system from Millipore (Burlington, MA, USA).

### 2.3. Color Intensity (ABS_450_) Determination

Honey color intensity was assessed spectrophotometrically by the ABS_450_ method (absorbance at 450 nm, after diluting honey to 50% *w*/*v*). Approximately 25 g of honey was mixed with 50 mL of warm (45–50 °C) distilled water until fully dissolved. The honey solution was then filtered to remove any debris. The absorbance of the filtrate was measured at 450 nm and at 720 nm (as a reference for turbidity) using an ultraviolet-visible (UV-vis) spectrophotometer (PG Instruments Ltd., Leicestershire, UK). The color intensity of honey was calculated as the difference between the absorbance at 450 nm and 720 nm, where 450 nm corresponds to the wavelength at which pigments such as carotenoids and flavonoids strongly absorb, and 720 nm serves as a baseline correction for turbidity or light scattering [21]. This ABS_450_ index correlates with honey’s polyphenol content and antioxidant potential, as darker honeys often contain more pigments like flavonoids, carotenoids, or polymerized polyphenolics from the Maillard reaction [22]. In our study, the ABS_450_ values for all samples were recorded in milli-absorbance units (mAU) for consistency.

### 2.4. Determination of Total Phenolic Content (TPC)

The total phenolic content (TPC) was determined using a modified Folin–Ciocalteu colorimetric method, which is a standard assay for polyphenols. First, an ethanol extract of each honey was prepared: approximately 0.1 g of honey was dissolved in 10 mL of 70% ethanol (*v*/*v*) in a capped vial. The mixture was stirred (vortexed) for 30 min to ensure thorough extraction of phenolic compounds, then filtered to obtain a clear extract.

For the assay, 100 µL of each honey’s ethanolic extract was mixed with 1.7 mL of distilled water in a test tube. Then 200 µL of Folin–Ciocalteu reagent (diluted 1:10 with water) was added. After 5 min, 2 mL of 7.5% Na_2_CO_3_ solution was added to alkaline-adjust the mixture. The reaction mixture was incubated at room temperature for 2 h in the dark to allow phenolic compounds to reduce the Folin–Ciocalteu reagent, resulting in a blue chromophore. Absorbance was measured at 765 nm against a blank containing 70% ethanol instead of the sample. Gallic acid was used for calibration, and a standard curve was constructed (linear equation: *y* = 0.0027*x* + 0.0261; *R*^2^ = 0.9916). Total phenolic content (TPC) was expressed as milligrams of gallic acid equivalents per 100 g of honey (mg GAE/100 g). Each sample was analyzed in triplicate, and the average values were reported [23,24].

### 2.5. HPLC-DAD-ESI+ Phenolic Compounds Method

Honey samples were extracted using acidified water (pH 2, adjusted with 1N HCl). Briefly, 1 g of honey was weighed into a 15 mL centrifuge tube, and 5 mL of pH 2 water was added. The mixture was vortexed for 1 min (Heidolph Reax Top, Heidolph Instruments GmbH & Co. KG, Schwabach, Germany), sonicated for 15 min in a sonicating bath (Elmasonic E 15 H, Elma Schmidbauer GmbH, Singen, Germany), and centrifuged at 10,000 rpm for 10 min at 24 °C (Eppendorf AG 5804, Eppendorf AG, Hamburg, Germany). The resulting supernatant was purified using a C18 solid-phase extraction column (Waters Sep-Pak Vac 6 cc, 1 g, Waters Corporation, Milford, MA, USA). The SPE column was conditioned with 3 mL methanol, then with 3 mL water and the extract were passed through the column, and the phenolic compounds were eluted with 1.5 mL methanol 90%. The elution operation was repeated 3 times, and the extracts were pooled. The final extract was filter looked through a Chromafil Xtra nylon 0.45 µm filter (Macherey-Nagel GmbH & Co. KG, Düren, Germany) and 20 µL was injected into the HPLC system.

Polyphenolic compounds were separated and identified using an Agilent 1200 HPLC system (Agilent Technologies, Santa Clara, CA, USA), equipped with a quaternary pump, autosampler, degasser, and a diode array detector (DAD), connected to a single quadrupole mass spectrometer (Agilent 6110 model). The chromatographic separation was carried out on a Kinetex XB-C18 column (4.6 × 150 mm, 5 µm; Phenomenex, Torrance, CA, USA). The mobile phases consisted of (A) water with 0.1% acetic acid and (B) acetonitrile with 0.1% acetic acid, using a gradient elution over 30 min (Table 3). The column was maintained at 25 °C with a constant flow rate of 0.5 mL/min.

Quantification of phenolic compounds was carried out using external calibration curves constructed from four commercially available standards: gallic acid (hydroxybenzoic acid), chlorogenic acid (hydroxycinnamic acid), luteolin (flavone), and rutin (flavonol). All standards were of analytical grade (≥98% purity) and sourced from Sigma-Aldrich (St. Louis, MO, USA) or Merck (Darmstadt, Germany). Calibration curves were established in methanol at five concentration levels, yielding coefficients of determination (*R*^2^) ranging between 0.9937 and 0.9981. Limits of detection (LOD) varied between 0.21 and 0.41 µg/mL, while limits of quantification (LOQ) ranged from 0.84 to 1.64 µg/mL.

For compounds for which reference standards were not commercially available, semi-quantitative determination was conducted by expressing concentrations in equivalents of structurally related standards (e.g., hydroxybenzoic acids as gallic acid equivalents, hydroxycinnamic acids as chlorogenic acid equivalents, flavones as luteolin equivalents, and flavonols as rutin equivalents).

Spectral data were collected in the 200–600 nm range for all detected peaks. Chromatograms were monitored at two key wavelengths: λ = 280 nm (for phenolic acids and flavanols) and λ = 340 nm (for flavonols and flavones). For mass spectrometry, full-scan data were acquired using positive ionization electrospray ionization (ESI^+^) mode, under the following conditions: capillary voltage 3000 V, source temperature 350 °C, nitrogen gas flow 7 L/min, and mass range *m*/*z* 120–1200. Data acquisition and interpretation were carried out using Agilent ChemStation software, version Rev B.02.01-SR2 [25].

### 2.6. Antioxidant Activity (DPPH, FRAP, ABTS Assays)

The antioxidant capacity was assessed using three complementary spectrophotometric assays 2.2, diphenyl-picryl-hydrazyl (DPPH) radical scavenging, ferric reducing antioxidant power (FRAP), and 2,2′-azinobis-(3-ethylbenzthiazolin-6-sulfonic acid) (ABTS) radical cation decolorization, which are widely applied in food and honey research. These methods, representing electron transfer (ET)-based mechanisms, are part of the most commonly standardized procedures for evaluating antioxidant potential in foods and dietary supplements [26]. Their combined application provides a broader perspective on radical scavenging and reducing capacity, compensating for the limitations of any single assay. For these assays, an ethanolic extract of each honey was prepared (as described above in Section 2.3) by dissolving 0.1 g honey in 10 mL of 70% ethanol. These extracts were used as the test solutions in each antioxidant assay. All antioxidant tests were conducted in triplicate for each sample to ensure reliability.

The DPPH test measures the ability of antioxidants in the sample to quench the stable free radical DPPH, which has a deep purple color. We followed a standard protocol with slight modifications [25]. In a cuvette, 100 µL of honey extract was mixed with 2.8 mL of 80 µM DPPH solution (in methanol). The mixture was incubated in the dark at room temperature for exactly 60 min. The decrease in absorbance at 517 nm was then recorded (DPPH has an absorption maximum at 517 nm, which diminishes upon reduction by an antioxidant). A control sample (A_0_) was prepared with 100 µL of 70% ethanol (no honey) in 2.8 mL DPPH solution. The percentage of DPPH radical scavenged by the sample was calculated according to Equation (1), as follows:

% Radical Scavenging Activity = [(A_0_ − A_1_)/A_0_] × 100 (1)

where A_0_ is the absorbance of the DPPH solution without sample (control) and A_1_ is the absorbance in the presence of the honey extract. Results were also expressed as micromoles of Trolox equivalents (µmol TE) per 100 g of honey.

The FRAP analysis evaluates the ability of the sample to reduce Fe^3+^ (ferric) to Fe^2+^ (ferrous) in the presence of TPTZ (2,4,6-tripyridyl-s-triazine), forming a blue Fe^2+^-TPTZ complex measured at 593 nm. We prepared a FRAP working solution fresh (containing acetate buffer, TPTZ, and FeCl_3_). In a test tube, 100 µL of honey extract was added to 0.5 mL of FRAP reagent. The mixture was incubated in the dark for 1 h at room temperature. Absorbance was then read at 593 nm. The results were expressed as µmol TE/100 g [25].

ABTS method involves the pre-formed ABTS^+^ radical cation (2,2′-azino-bis(3-ethylbenzothiazoline-6-sulfonic acid)) which has a green-blue color. The ABTS^+^ solution was generated by reacting ABTS with potassium persulfate and allowing it to incubate in the dark (12–16 h). The stock ABTS^+^ was then diluted with phosphate-buffered saline to an absorbance of 0.70 (±0.02) at 734 nm. For the assay, 100 µL of honey extract was mixed with 2.9 mL of the diluted ABTS^+^ solution. After exactly 1 min of reaction (since decolorization can be rapid with strong antioxidants), the absorbance at 734 nm was recorded. Trolox was again used as the standard, and results were reported as µmol TE/100 g [25].

### 2.7. Kinetic Analysis of DPPH Radical Scavenging

To further characterize the dynamics of the antioxidant activity of the honeys, we performed a kinetic analysis of the DPPH scavenging over time. Among the three antioxidant assays used in this study (DPPH, ABTS, FRAP), only DPPH was selected for kinetic modeling due to its compatibility with time-resolved measurements under stable assay conditions. Unlike ABTS and FRAP, which are typically single-point or endpoint assays, the DPPH method enables dynamic monitoring, making it uniquely suited for kinetic analysis. Instead of the single time-point (60 min) used in the standard DPPH test, we measured the DPPH inhibition at multiple time intervals, 30, 60, 90, and 120 min after mixing the honey extract with DPPH. These time-course data were then fitted to kinetic models of zero-order ([*A*]*t* = [*A*]0 − *k·t*), first-order (*ln*[*A*]*t* = *ln*[*A*]0 − *k·t*), and second order (1/[*A*]*t* = 1/[*A*]0 + *k·t*) to determine which best described the reaction. In these equations, [*A*]*t*, represents the percentage of DPPH remaining (or conversely, % DPPH inhibited) at time *t*, [*A*]0 is the initial % DPPH (at 30 min as a baseline), and *k* is the reaction rate constant.

For each honey sample, we transformed the experimental DPPH inhibition data according to each model (i.e., plotting [*A*] vs. *t*, ln[*A*] vs. *t*, and 1/[*A*] vs. *t*) and performed linear regression, using Microsoft Excel, to see which plot gave the best linear fit (highest *R*^2^). The model with the highest correlation coefficient *R*^2^ was considered the best fit for that sample’s kinetics. From the best-fit model (in our case, predominantly zero-order), we obtained the slope of the linear regression which corresponds to the rate constant k (with units % DPPH inhibited per minute). This k value indicates how fast the honey’s antioxidants neutralize the DPPH radicals. We compared k among samples to rank their reaction speeds.

Additionally, we calculated the half-life (t_1/2_) for the DPPH reaction in each sample. For a zero-order reaction, half-life is given by *T*_1/2_= [*A*]_0_/2*k*. This represents the time required for the initial DPPH (at 30 min) to be reduced by half in the presence of the honey antioxidants. Finally, we determined the area under the curve (AUC) for the DPPH inhibition vs. time plot for each honey as an integrative measure of total antioxidant effect over the 2 h period. AUC was calculated using the trapezoidal numerical integration method, with units of DPPH%/min. A higher AUC indicates a greater overall scavenging effect sustained over time [27].

### 2.8. Principal Component Analysis

Principal Component Analysis (PCA) was employed as an exploratory multivariate technique to investigate patterns in antioxidant behavior and phenolic composition across honey samples. A composite dataset was constructed from color intensity (ABS_450_), antioxidant activities (DPPH, ABTS, FRAP), and HPLC-identified phenolic compounds present above 1 mg/100 g. All variables were mean-centered and scaled to unit variance, and PCA was performed on the correlation matrix to prevent dominance of variables with larger ranges. The main PCA model was complemented by two additional exploratory models: one including only spectrophotometric parameters (ABS_450_, DPPH, ABTS, FRAP), and another restricted to HPLC-derived phenolic subclasses (total phenolic acids, total flavonoids, and individual compounds > 1 mg/100 g). These parallel approaches allowed us to compare the discriminative potential of colorimetric versus chromatographic data.

Although the dataset was limited (n = 8), PCA served as a useful preliminary tool for visualizing clustering trends and identifying the key variables driving differences among honeys. Similar applications in foodomics and metabolomics support its appropriateness in small but multifactorial datasets [28,29,30].

### 2.9. Statistical Analysis

One-way analysis of variance (ANOVA) was applied to assess significant differences among honey samples for each measured parameter. When significant effects were detected (*p* < 0.05), Tukey’s HSD post hoc test was used to identify specific differences between groups. Results are provided as mean ± standard deviation (SD).

Pearson correlation coefficients (r) were calculated to explore relationships between phenolic composition, color intensity (ABS_450_), and antioxidant capacity (DPPH, ABTS, FRAP). All statistical analyses were conducted using Microsoft Excel (v. 2021) and JASP (0.19.3) [31] for basic descriptive statistics and regression analysis, and MATLAB (R2023a, MathWorks, Natick, MA, USA) [28] for multivariate analysis (Pearson correlation and PCA). A significance threshold of *p* < 0.05 was applied throughout.

## 3. Results

### 3.1. Color Intensity (ABS_450_)

**Figure 1:**
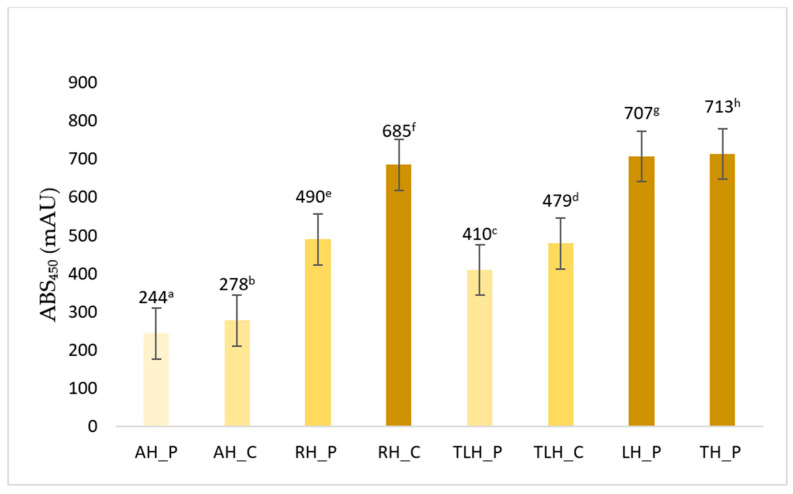
Color intensity (ABS450, mAU) of different honey samples measured at 50% *w*/*v* dilution. Data are presented as mean ± standard deviation (n = 3). Distinct superscript letters denote statistically significant differences between samples (*p* < 0.05). Sample codes are defined as follows: AH_P—acacia honey from producer; AH_C—acacia honey from commercial source; RH_P—rapeseed honey from producer; RH_C—rapeseed honey from commercial source; TLH_P—linden honey from producer; TLH_C—linden honey from commercial source; LH_P—lavender honey from producer; TH_P—thyme honey from producer.

The tested honey samples showed notable variation in color intensity, with ABS_450_ values ranging from 244 mAU (lightest) to 713 mAU (darkest). As illustrated in Figure 1, acacia honey from the local producer (AH_P) exhibited the lowest color intensity (244 mAU), consistent with its nearly transparent, pale golden appearance. The commercial acacia sample (AH_C) showed a slightly higher value (278 mAU) but remained visually similar.

Rapeseed honeys showed more pronounced variation. The producer sample (RH_P) recorded a moderate 490 mAU, while the commercial counterpart (RH_C) reached 685 mAU, marking a visible shift toward an amber hue. A similar trend was observed in linden honey, where the producer sample (TLH_P) had an ABS_450_ of 410 mAU (light amber), compared to 479 mAU for the commercial sample (TLH_C). Among all, lavender honey (LH_P) and thyme honey (TH_P) stood out as the darkest, with absorbance values of 707 mAU and 713 mAU, respectively. Both displayed deep amber to brown coloration, typical of honeys rich in phenolic pigments.

Statistical analysis indicated that each sample’s ABS_450_ value differed significantly from the others (*p* < 0.05), as indicated by distinct superscript letters in Figure 1. This suggests that botanical origin and source type (producer vs. commercial) play a substantial role in determining color intensity.

### 3.2. Total Phenolic Content

The total phenolic content (TPC), determined by the Folin–Ciocalteu method, exhibited considerable variation depending on both botanical origin and source (producer vs. commercial), as shown in Table 4. The results ranged from 179.26 ± 23.57 mg GAE/100 g in the producer acacia honey (AH_P) to a maximum of 586.67 ± 18.33 mg GAE/100 g in the linden honey commercial (TLH_C). Statistical analysis revealed significant differences (*p* < 0.05) between several groups. RH_P and LH_P formed a separate group, significantly different from TLH_C and TH_P, which had the highest TPC values.

### 3.3. Identification of Phenolic Compounds

Seventeen individual phenolic compounds were identified across the honey samples, comprising both phenolic acids and flavonoids (Table 5). Among the phenolic acids, common constituents such as gallic acid and chlorogenic acid were detected in several samples, while others appeared more selectively. In the flavonoid group, rutin (a glycoside of quercetin) was consistently present, along with other flavonol and flavone derivatives, either in glycosylated form or as aglycones.

**Figure 2:**
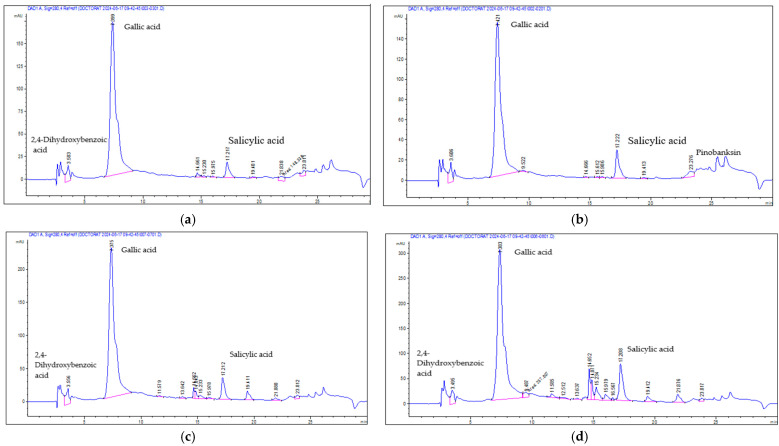

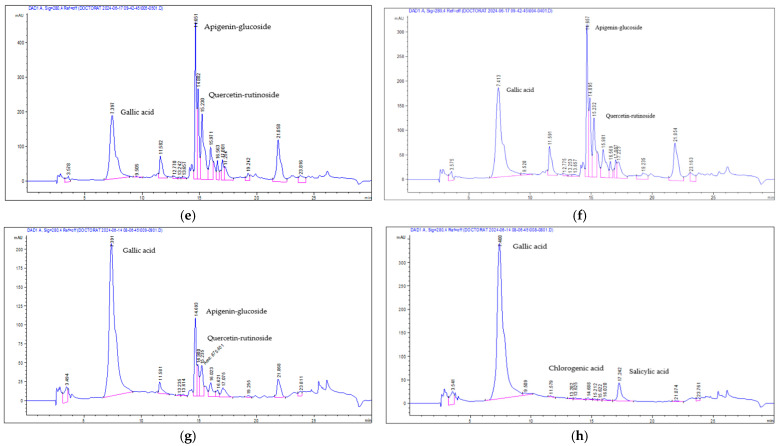
HPLC chromatograms at 340 nm for: (**a**) AH_P—acacia honey from producer; (**b**) AH_C—acacia honey from commercial source; (**c**) RH_P—rapeseed honey from producer; (**d**) RH_C—rapeseed honey from commercial source; (**e**) TLH_P—linden honey from producer; (**f**) TLH_C—linden honey from commercial source; (**g**) LH_P—lavender honey from producer; (**h**) TH_P—thyme honey from producer.

Figure 2a–h consisted of the HPLC chromatograms at 340 nm for each honey sample, where distinct profiles were observed. Linden honey showed prominent peaks corresponding to flavonoid glycosides (like rutin and quercetin glycoside) (Figure 2e,f), whereas thyme honey’s chromatogram had a dominant gallic acid peak and other phenolic acids (Figure 2h).

### 3.4. Quantitative Phenolic Profiles

The phenolic profiles varied significantly depending on both botanical origin and source, with nearly all compounds showing statistically significant differences (*p* < 0.05). Among the phenolic acids, gallic acid was the most abundant across samples and showed remarkable differences. The highest concentration was found in thyme honey (TH_P, 127.49 ± 5.69 mg/100 g), significantly exceeding all other honeys (superscript g), followed by RH_C (rapeseed, commercial) and LH_P (lavender), which also stood out with elevated levels (f and d, respectively). In contrast, acacia honeys (AH_C and AH_P) recorded the lowest concentrations (a and b), aligning with their generally mild phytochemical profiles. Chlorogenic acid was almost exclusively associated with linden honeys, with TLH_P and TLH_C containing significantly higher levels (26.43 and 23.92 mg/100 g, f and e) than all other samples. This compound was nearly absent in acacia and thyme honeys. When considering salicylic acid, the highest concentration was observed in RH_C (22.70 mg/100 g, g), significantly above both producer and commercial samples of other botanical types. Lavender honey (LH_P) showed no detectable salicylic acid, a notable absence that distinguishes it from the rest.

Flavonoid analysis revealed even sharper contrasts. TLH_P (linden, producer) exhibited a rich flavonoid profile, with a total of 233.31 mg/100 g, significantly higher than any other sample (e). This was driven largely by high amounts of rutin (70.5 mg, e) and galangin-glucoside (57.47 mg, f). The corresponding commercial linden sample (TLH_C) followed, but with significantly lower flavonoid content (149.43 mg/100 g, d). By contrast, acacia honeys (AH_P and AH_C) contained the lowest flavonoid levels overall (7.82 and 6.21 mg/100 g, a), with no significant difference between the two. Apigenin derivatives (glucoside and apiosyl-glucoside forms) were predominantly present in linden and rapeseed honeys, with TLH_P again showing the highest values (29.46 and 19.29 mg/100 g). These compounds were absent in acacia honeys and thyme, adding to the distinctiveness of the linden profile.

Looking at total sums, TH_P (thyme) emerged as the richest source of phenolic acids, totaling 154.57 mg/100 g (f), followed closely by RH_C (132.99 mg, e) and TLH_P (112.18 mg, d). However, when flavonoids were included, TLH_P dominated in overall phenolics (345.49 mg/100 g, h), far surpassing even the second highest (TLH_C, 261.67 mg, g). Table 6 summarizes the quantitative profile of total polyphenols as determined by HPLC-DAD-ESI^+^ for all 8 types of honey.

As expected, TPC values obtained via Folin–Ciocalteu were consistently higher than those by HPLC, reflecting the method’s response to all reducing substances, not solely identifiable phenolics. The TLH_C sample reached 586.67 ± 18.33 mg GAE/100 g by Folin, compared to 261.67 ± 4.1 mg/100 g by HPLC. In most cases, commercial samples (AH_C, TLH_C) showed higher TPC values than those from local producers (AH_P, TLH_P), but this trend was not consistently observed in the HPLC results. This highlights that colorimetric methods may overestimate phenolic content due to interfering compounds, while HPLC provides a more accurate assessment of true phenolic composition.

Taken together, the chromatographic data underline the strong variability of phenolic composition among the analyzed honeys, with clear dominance of phenolic acids in thyme and rapeseed samples, contrasted by the flavonoid-rich profile of linden honey. In contrast, acacia honey consistently exhibited the lowest concentrations across both phenolic acids and flavonoids. These differences highlight the distinct chemical signatures of each floral type.

### 3.5. Antioxidants Capacity

To evaluate the functional implications of these compositional differences, the antioxidant activity of the honey samples was determined using DPPH, ABTS, and FRAP assays. The results showed marked differences across samples, strongly influenced by botanical origin and, in some cases, by supply source (producer vs. commercial). Most comparisons yielded statistically significant differences (*p* < 0.05). In the DPPH assay, thyme honey (TH_P) exhibited the highest radical scavenging capacity (21.82 µmol T/100 g, superscript c), significantly above all other samples. Linden honeys (TLH_P, TLH_C) also showed elevated DPPH values (20.01 and 19.11, b,c and b, respectively), while the lowest values were recorded in acacia honeys (AH_P and AH_C, 16.82 and 19.60, a and b), confirming their generally modest antioxidant profile.

The ABTS assay highlighted more clear distinctions. Again, TH_P showed the strongest activity by far (145.72 µmol T/100 g,f), significantly higher than all other samples. Linden honeys followed, with TLH_P (107.03) and TLH_C (91.55) showing robust capacity (e and d), whereas acacia samples (AH_P, AH_C) remained at the lowest end (approximative 8 µmol, a), without significant difference between them. RH_C also demonstrated a high ABTS value (68.34, c), suggesting enhanced antioxidant potential in the commercial rapeseed honey, possibly due to a broader phenolic profile or blending.

In the FRAP assay, which reflects reducing power, TH_P and RH_C shared the highest value (97.55 µmol T/100 f), indicating strong electron-donating potential. Linden (TLH_P, 77.07, e; TLH_C, 67.81, d) and lavender (LH_P, 70.46, d,e) also exhibited high FRAP responses. The lowest reducing activity was again seen in AH_P (13.09, a).

Overall, thyme and linden honeys consistently appeared to exhibit the strongest antioxidant responses across all three assays, while acacia honeys showed the lowest values, with rapeseed and lavender occupying an intermediate position. These results establish a clear gradient in antioxidant potential among the studied honeys. Detailed numerical values for DPPH, ABTS, and FRAP measurements are summarized in Table 7.

### 3.6. Correlation Between Phenolic Composition, Color, and Antioxidant Activity

The Pearson correlation matrix revealed distinct linear relationships among the analyzed variables. Among the strongest positive correlations were those observed between gallic acid and the parameters total phenolic acid (r = 0.83), FRAP (r = 0.75) and ABS450 (r = 0.80), suggesting that gallic acid is one of the main contributors to the overall antioxidant potential and color intensity. Similarly, 2,4-dihydroxybenzoic acid showed a close association with the same parameters (total phenolic acid r = 0.77, FRAP r = 0.73, ABS450 r = 0.83). Total phenolic acid, calculated as the sum of individual phenolic compounds detected by HPLC, showed strong correlations with ABTS (r = 0.86) and FRAP (r = 0.91).

Regarding flavonoids, quercetin-glucoside, apigenin-glucoside and quercetin-rutinoside showed very strong correlations with each other (r > 0.95), reflecting their possible co-occurrence in the analyzed honey types and similar chemical behavior. However, their correlations with antioxidant parameters were weak to moderate (r between 0.15–0.27). In line with this, total flavonoids quantified by HPLC displayed only low correlations with antioxidant parameters (DPPH r = 0.21; ABTS r = 0.25) compared with total phenolic acids.

**Figure 3:**
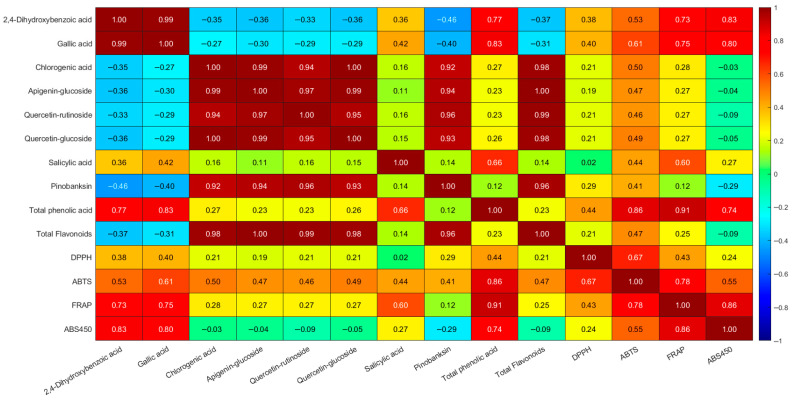
Pearson correlation matrix between phenolic compounds identified by HPLC (concentration > 1 mg/100 g), total phenolic acids, total flavonoids, color intensity (ABS_450_), and antioxidant activity parameters (DPPH, ABTS, FRAP). Pearson’s correlation coefficient (r) ranges from −1 to +1, indicating the strength and direction of the linear relationship between variables. Strong positive correlations (r ≥ 0.71) were represented in dark red, while strong negative correlations (r ≤ −0.71) appeared in dark blue. Moderate positive correlations were shown in shades of orange to yellow, whereas moderate negative correlations were represented in light bluish tones. Weak or no correlations (r between −0.30 and 0.30) appeared in light green.

Honey color, expressed as ABS_450_, was strongly associated with total phenolic acids (r = 0.74), FRAP (r = 0.86), and with individual compounds such as gallic acid and 2,4-dihydroxybenzoic acid. These results indicate that phenolic acids contributed more consistently than flavonoids to the measured antioxidant responses. Full correlation coefficients are presented in Figure 3.

### 3.7. Kinetic Modeling of DPPH Scavenging Activity

**Figure 4:**
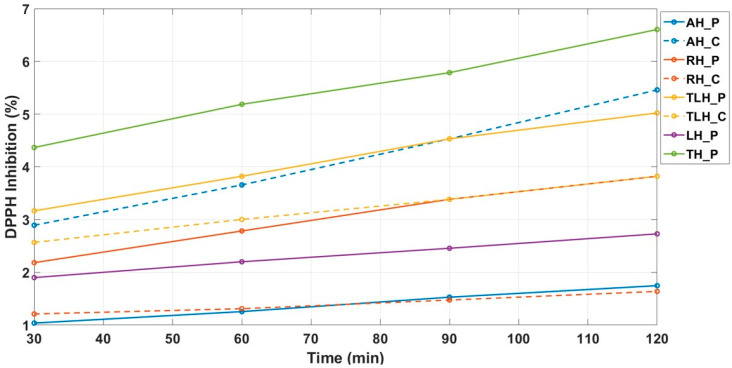
Time-dependent evolution of DPPH radical scavenging activity (%); AH_P—acacia honey from producer; AH_C—acacia honey from commercial source; RH_P—rapeseed honey from producer; RH_C—rapeseed honey from commercial source; TLH_P—linden honey from producer; TLH_C—linden honey from commercial source; LH_P—lavender honey from producer; TH_P—thyme honey from producer.

To further characterize the antioxidant responses, the kinetics of DPPH radical scavenging were monitored over a two-hour period. The kinetic evaluation showed that all honey samples followed a predominantly zero-order reaction model, characterized by linear reaction progress with respect to time and *R*^2^ values exceeding 0.98 in all cases (Table 8 and Figure 4). This high degree of linearity supports the hypothesis that antioxidant components act in a sustained, time-independent manner during the initial 2 h window. In zero-order kinetics, the rate of radical scavenging remains constant, likely reflecting a steady release or reactivity of antioxidant molecules.

**Figure 5:**
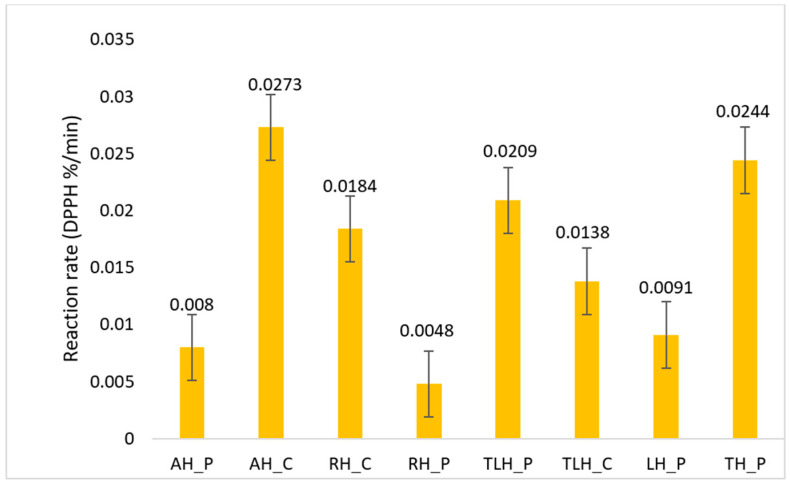
Antioxidant reaction rate of different honey types (DPPH %/min); AH_P—acacia honey from producer; AH_C—acacia honey from commercial source; RH_P—rapeseed honey from producer; RH_C—rapeseed honey from commercial source; TLH_P—linden honey from producer; TLH_C—linden honey from commercial source; LH_P—lavender honey from producer; TH_P—thyme honey from producer.

The reaction rate constant (*k*), derived from the slope of the zero-order model, varied significantly across samples. The fastest radical neutralization was observed in AH_C (k = 0.0273%/min), followed by TH_P (0.0244%/min) and TLH_P (0.0209%/min), indicating a rapid initial antioxidant response. In contrast, RH_P displayed the lowest rate (0.0048%/min), suggesting slower-reacting antioxidant constituents. These differences are visualized in Figure 5.

Complementary to rate, the half-life (t_1_/_2_) of the reaction, defined as the time required to achieve half of the total DPPH inhibition, ranged from 52.98 min (AH_C) to 126.04 min (RH_P), with faster-reacting honeys reaching their effective antioxidant potential more rapidly.

To assess overall antioxidant performance, the area under the curve (AUC) for DPPH inhibition over time was calculated (Table 9). AH_P exhibited the highest AUC (493.7 DPPH·min), despite its moderate reaction rate. This suggests that while some honeys (e.g., AH_C, TH_P) act quickly, others (e.g., AH_P) sustain their antioxidant activity over time. High AUC values thus indicate not only immediate efficacy but also durability of antioxidant effect.

### 3.8. Principal Component Analysis (PCA)

To integrate the compositional and functional data, a principal component analysis (PCA) was performed using phenolic compounds, antioxidant assay results (DPPH, ABTS, FRAP), kinetic parameters, and color intensity as input variables. The model explained 85.0% of the total variance, with PC1 accounting for 47.0% and PC2 for 38.0%.

The biplot revealed a clear clustering of samples according to floral origin. Thyme honey (TH_P) was positioned on the positive side of PC2, close to gallic acid, 2,4-dihydroxybenzoic acid, and ABS_450_, reflecting its dominance in phenolic acids and intense pigmentation. Linden honeys (TLH_P and TLH_C) grouped on the positive side of PC1, in close association with flavonoids (rutin, quercetin derivatives, apigenin glucosides) and chlorogenic acid, consistent with their higher flavonoid content and stronger reducing power. Rapeseed honeys (RH_P and RH_C) occupied intermediate positions: RH_C aligned more closely with phenolic acid vectors and antioxidant assays, while RH_P diverged along PC2, suggesting compositional variability between producer and commercial sources. Acacia honeys (AH_P and AH_C) clustered on the negative side of PC1, distant from most phenolic and antioxidant variables, in agreement with their lower phenolic content and weaker antioxidant scores. Lavender honey (LH_P) was located independently, without strong association to any single variable group, suggesting a more intermediate profile.

**Figure 6:**
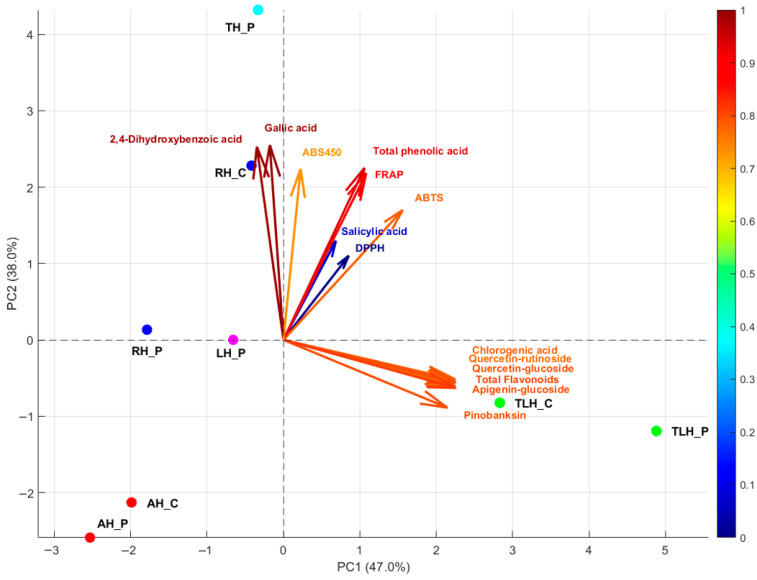
Principal component analysis biplot of honey samples integrating phenolic compounds, antioxidant assays, and color intensity; AH_P—acacia honey from producer; AH_C—acacia honey from commercial source; RH_P—rapeseed honey from producer; RH_C—rapeseed honey from commercial source; TLH_P—linden honey from producer; TLH_C—linden honey from commercial source; LH_P—lavender honey from producer; TH_P—thyme honey from producer. The color scale on the right represents variable contribution to the PCA model.

Regarding supply chain, acacia and linden honeys showed similar positions for producer and commercial samples, whereas rapeseed honeys displayed more divergence, particularly along PC2. For lavender and thyme, where only producer samples were available, their distinct positions emphasized unique chemical signatures, reinforcing their potential use in authentication (Figure 6).

Overall, the PCA results highlight the major compositional gradients among the studied honeys and demonstrate the influence of both botanical origin and, to a lesser extent, supply source.

## 4. Discussion

Understanding the functional potential of honey requires more than a general appreciation of its sweetness or floral aroma. While traditional uses have long highlighted its role in food preservation and household remedies, advances in modern biochemistry have more recently deepened our insight of the mechanisms underlying these properties. Among the most relevant contributors are phenolic compounds, natural antioxidants that play a central role in neutralizing free radicals and modulating oxidative processes. In this study, we extend the previous work on Romanian monofloral honeys [20] by providing a more detailed analysis of their phenolic composition and antioxidant behavior. Using samples from five floral origins and two supply chains, we observed clear differences in phenolic load and antioxidant responses, shaped primarily by botanical origin and, to a lesser extent, by whether the samples were sourced directly from producers or obtained through retail channels.

The visual appearance of honey, particularly its color intensity, is one of the most immediately noticeable and functionally informative attributes. It reflects not only the floral source but also a range of environmental and processing factors, including nectar composition, temperature, storage duration, and even handling methods during acquisition and bottling [29]. In this study, the color intensity of the honey samples, assessed via absorbance at 450 nm (ABS_450_), showed substantial variation, from the nearly colorless acacia honey to the deeply pigmented thyme and lavender varieties. These differences are largely attributed to the presence of flavonoids, carotenoids, and other phenolic pigments, which are known to contribute both to color and to antioxidant capacity [30,32]. Our results align with previous research indicating that darker honeys, such as buckwheat, heather, or manuka, tend to contain higher levels of phenolic compounds and may exhibit greater antioxidant potential than lighter types like acacia or citrus honeys [33,34].

Differences in ABS_450_ values between producer and commercial honeys, especially for rapeseed and linden, may reflect not only floral origin but also processing-related influences (heating, blending, storage), which can alter pigment stability and antioxidant properties [35]. Thus, color is a useful but non-specific indicator that reflects both botanical origin and technological influences. Moreover, color is not just a biochemical indicator but also a major factor influencing consumer perception and marketability. Preferences differ geographically. While European consumers often associate darker, amber-toned honeys with richer flavor and higher quality, North American preferences tend toward lighter-colored honeys with milder taste profiles. These cultural and sensory expectations reinforce the importance of visual parameters in both scientific evaluation and commercial positioning of honey [36].

At the compositional level, botanical origin appeared to be the main determinant. Seventeen phenolic compounds were identified across the analyzed samples; however, not all markers commonly reported for the respective monofloral honeys were detected. For instance, quercetin and pinocembrin, previously described in acacia and linden honeys [23,37,38] were either absent or detected at very low levels. Similar inconsistencies have also been noted in comparative studies from Turkey [39] and Poland [37], suggesting that environmental and geographical factors may influence phenolic composition. The absence of these markers in the present samples may be attributed to such factors, as well as limited size of the dataset. Linden honey was rich in flavonol glycosides such as rutin and quercetin derivatives, while thyme honey was characterized by higher levels of phenolic acids, especially gallic acid. These findings are generally consistent with reports identifying rutin as a chemotaxonomic marker for linden honey and gallic acid as a key constituent of thyme honeys from Mediterranean regions [40,41,42]. Such distinctions may support the concept of floral-specific phenolic fingerprints. In addition, chlorogenic acid was most abundant in linden honeys (23.9–26.4 mg/100 g), showing significantly higher levels than in lavender, rapeseed, acacia and thyme honeys, where only trace amounts (<7 mg/100 g) were detected. This distribution suggests its potential use as a chemotaxonomic markers for linden honey, consistent with reports on other European Tilia honeys [43].

The highest total phenolic contents were observed in linden (586.67 mg GAE/100 g, TLH_C) and thyme (562.59 mg GAE/100 g, TH_P), supporting prior reports of these types as phytochemical-rich honeys [23,44,45]. In contrast, acacia consistently showed the lowest values. Differences between producer and commercial honeys were not systematic. While commercial acacia and rapeseed samples generally contained fewer phenolics, commercial linden honey displayed the highest overall levels. This may suggests that processing does not uniformly reduce phenolic compounds and could potentially preserve or release bound forms under certain conditions [46,47].

It is worth noting that different analytical approaches provided complementary perspectives. Folin–Ciocalteu assays, for instance, yielded higher TPC values than HPLC, since the reagent reacts not only with phenolics but also with other reducing substances such as ascorbic acid, amino acids, and Maillard reaction products. This explains why some commercial samples appeared richer in phenolics by Folin–Ciocalteu but did not consistently show the same trend in chromatographic profiling. Such discrepancies underline the need to interpret spectrophotometric data cautiously and in conjunction with compound-specific methods [24,48].

Compound-level differences further illustrated the diversity of phenolic profiles. Linden honey contained rutin (up to 70 mg/100 g), quercetin derivatives, galangin, and chlorogenic acid, while thyme honey was especially rich in gallic acid (127 mg/100 g). Other flavonoids such as pinocembrin, kaempferol, and apigenin were present in smaller amounts but are recognized in literature as contributors to honey’s antioxidant performance [49]. Salicylic acid was detected in several honeys, including acacia, linden, and thyme, but the highest concentration was observed in rapeseed honey, particularly in the commercial sample (22.7 mg/100 g). This elevated level may distinguish rapeseed from the other floral types, where concentrations ranged between 4.8 and 12.6 mg/100 g, while lavender honey contained no detectable salicylic acid. The marked abundance in rapeseed could reflect contributions from Brassicaceae-derived metabolites and/or propolis-derived components. Studies on honey enrichment and propolis composition show that bee-product additions elevate phenolic loads and antioxidant responses [48], and salicylic acid has also been reported as a component of propolis extracts [50]. Together, these observations suggest that HPLC profiling can provide insight into potential chemotaxonomic markers such as gallic acid in thyme and rutin and chlorogenic acid in linden. Such compound-level markers have been increasingly emphasized in metabolomics studies as reliable indicators of floral origin and authenticity [51,52]. Several studies have proposed specific phenolic compounds as indicators of floral origin in honey, including rutin and quercetin in linden [9,23,53], gallic and caffeic acids in thyme [52,54], and pinobanksin derivatives in acacia [43,55]. While some of these compounds were identified in the present samples, such as rutin in linden and gallic acid in thyme, others, including quercetin or pinocembrin, were either absent or present at trace levels. These discrepancies highlight the complexity of using phenolic markers for botanical authentication, especially in studies with limited sample sizes. The partial overlap between our findings and those reported in broader surveys [56,57,58] suggest that floral origin alone does not fully determine phenolic composition, and that environmental, seasonal, and geographical factors likely play a significant role. Indeed, Romanian [23] and Southeastern European [43,52,59] studies have shown that compounds considered typical in one region may be underrepresented elsewhere, even within the same floral type. Therefore, caution is needed when interpreting phenolic profiles in monofloral honeys, particularly in exploratory studies, and future work should prioritize larger, regionally stratified datasets to strengthen the diagnostic value of such chemotaxonomic indicators.

Antioxidant activity, measured by DPPH, ABTS, and FRAP assays, showed marked variation across floral types. Thyme and linden honeys consistently exhibited the strongest activity, particularly in ABTS and FRAP, while acacia honeys had the weakest performance. Rapeseed and lavender honeys occupied an intermediate position. These results reflect the distribution of phenolic compounds, reinforcing the link between polyphenol content and antioxidant capacity, in agreement with earlier studies [60,61].

Correlation analysis provided further insight into the relative contributions of phenolic subclasses The results indicated that phenolic acids such as gallic and 2,4-dihydroxybenzoic acids were the strongest contributors to FRAP, ABTS, and color intensity, whereas flavonoids showed weaker or moderate associations, suggesting that their role may be complementary or synergistic. This suggests that, in the honeys analyzed here, phenolic acids are the primary drivers of antioxidant activity, although flavonoids contribute synergistically. Gallic acid, reaching its maximum in thyme honey (127 mg/100 g), showed strong positive correlations with FRAP (r = 0.75), ABTS (r = 0.73), and color intensity (r = 0.80), underlining its role as a fast-acting, low-molecular-weight scavenger. Similarly, 2,4-dihydroxybenzoic acid contributed substantially to antioxidant potential, particularly in rapeseed and thyme honeys. In contrast, flavonoids such as rutin and quercetin derivatives, abundant in linden honey, correlated more with FRAP and ABS_450_ than with DPPH or ABTS. This suggests that flavonoids contribute mainly to reducing power and sustained antioxidant potential rather than to rapid radical quenching, consistent with broader reports on flavonoid-driven bioactivity [37,62]. This pattern explains why linden honeys performed strongly in FRAP but showed relatively lower ABTS responses compared with thyme honeys, despite their high flavonoid load.

Salicylic acid, detected at unusually high concentrations in commercial rapeseed honey (22.7 mg/100 g), may also have contributed to its disproportionately strong DPPH response relative to its moderate total phenolic content. While explicit links between salicylic acid and radical scavenging activity in honey have not yet been established, its detection at such high levels aligns with recent reports of diverse phenolic acids shaping honey’s antioxidant outcomes [63]. Conversely, lavender honey, although darkly pigmented, lacked salicylic acid and contained only moderate levels of gallic acid, which may account for its weaker-than-expected ABTS performance. Some exceptions, such as the commercial rapeseed sample (RH_C), which exhibited unexpectedly high DPPH activity despite moderate phenolic levels, highlight the role of non-phenolic antioxidants including enzymes, ascorbic acid, or Maillard reaction products [7,32]. Color intensity correlated positively with FRAP and TPC, supporting its role as a practical indicator of phenolic richness [18]. However, exceptions, like lavender honey, which despite its dark pigmentation showed only moderate ABTS scores, confirm that color alone cannot fully predict antioxidant potential. This observation aligns with chemometric studies showing that pigmentation and flavonoid load must be interpreted together with phenolic acid profiles to achieve reliable classification of honey bioactivity [7,64]. Overall, our findings emphasize that phenolic acids, particularly gallic and 2,4-dihydroxybenzoic acid, are the primary determinants of rapid radical scavenging in Romanian studied honeys, while flavonoids such as rutin and quercetin derivatives play complementary roles, strengthening reducing power and sustaining antioxidant activity. Such compound-specific insights, supported by HPLC profiling, may provide a stronger mechanistic basis for understanding how botanical origin dictates antioxidant performance.

To capture the time dimension of antioxidant responses, we extended the analysis of DPPH scavenging to multiple time points. This kinetic perspective revealed distinct patterns. Thyme and linden honeys combined strong and rapid scavenging with sustained activity, while acacia honey, though weaker overall, showed a stable kinetic profile with a relatively high area under the curve (AUC). These differences may reflect the coexistence of fast-acting antioxidants (e.g., enzymes, small phenolic acids) and slower, more persistent compounds such as flavonoids.

Kinetic modeling indicated a predominant zero-order response across samples, suggesting steady radical scavenging over the two-hour period. Such findings highlight that antioxidant performance is shaped not only by concentration but also by persistence, which may be relevant in contexts such as food preservation or gradual oxidative stress modulation. Such findings emphasize that antioxidant performance is shaped not only by concentration but also by persistence, parameters such as rate constant, half-life, and AUC offering a more comprehensive description of efficiency over time [27].

The phenolic composition determined by HPLC supported these kinetic observations. Thyme honey, characterized by its high content of gallic acid and other low-molecular-weight phenolic acids, exhibited rapid and sustained scavenging. Linden honey, enriched in flavonoid glycosides such as rutin, displayed a more gradual but persistent response. In contrast, the modest profile of acacia honey, with the lowest phenolic load and limited compound diversity, was reflected in its weaker kinetics. These results highlight the value of integrating kinetic modeling with compound-level analysis to better understand the mechanistic basis of antioxidant capacity in honey.

To further explore the relationships among phenolic composition, antioxidant activity, and color traits, we applied principal component analysis (PCA). After mean-centering and scaling variables to unit variance, the first two principal components explained nearly 85% of the total variance, providing a clear differentiation of honey types in the PCA space. PC1 (47.3% variance) reflected a general gradient of bioactive richness, with strong positive contributions from total phenolic content and antioxidant assays (DPPH, ABTS, FRAP). Thyme and linden honeys, with higher phenolic loads and stronger antioxidant capacity, were positioned toward the positive side of PC1, while acacia honeys, characterized by lower phenolic levels and weaker antioxidant scores, clustered on the negative side. This separation is consistent with earlier studies showing that phenolic-rich honeys such as buckwheat and heather cluster similarly in multivariate space due to their high antioxidant activity [61,62].

PC2 (38.0%) of variance) was driven mainly by color intensity (ABS_450_) and flavonoid content, with an opposite contribution from DPPH activity. Along this axis, darker and flavonoid-rich honeys, such as linden and thyme, were distinguished from lighter honeys like rapeseed, which nevertheless retained strong DPPH responses. These results suggest that rapid-acting, low-molecular-weight antioxidants (e.g., simple phenolic acids or enzymatic components) can drive DPPH activity independently of color or flavonoid content.

The PCA biplot also highlighted clear botanical signatures. Acacia samples grouped together near gallic acid, confirming their relatively simple phenolic profiles and modest antioxidant performance. Rapeseed honeys aligned with phenolic acid vectors and balanced antioxidant responses, while linden honeys clustered with FRAP and flavonoid vectors, reflecting their richness in rutin and related flavone glycosides [40]. Thyme honey appeared clearly separated along both PC1 and PC2, emphasizing its dense pigmentation and high content of phenolic acids such as gallic acid and pinobanksin derivatives.

Differences between producer and commercial samples were also visible. Acacia and linden honeys from both sources overlapped closely, suggesting compositional similarity across supply chains. In contrast, rapeseed honeys showed more pronounced divergence, particularly along PC2, pointing to possible variability linked to processing or storage. Lavender and thyme honeys, available only from producers, occupied distinct positions, reinforcing their potential as targets for traceability and authentication.

Overall, PCA captured the main compositional gradients underlying honey variability: PC1 reflected the global polyphenol, antioxidant axis, while PC2 emphasized the contribution of color intensity and flavonoid subclasses. By integrating PCA with HPLC- phenolic profiles, the analysis provides a stronger mechanistic explanation for the observed clustering, where individual compounds such as gallic acid, rutin, and salicylic acid acted as drivers of botanical differentiation. Similar outcomes have been reported, showing that phenolic fingerprints obtained by HPLC may be strong determinants of clustering patterns in honeys of different floral origins [32,65].

These exploratory results align with previous multivariate analyses of European honeys [61,62,66] and are in agreement with broader applications of PCA in foodomics and metabolomics, where such unsupervised techniques are widely used to identify clustering patterns and compositional drivers, even in small datasets [67,68]. While different extraction solvents were used across assays, each method followed established protocols optimized for the target compounds. Prior to multivariate analysis, all datasets were standardized to enhance comparability. Even so, differences in sample preparation may affect direct correlations, and results should be interpreted as exploratory.

While this study offers a detailed characterization of the antioxidant capacity and phenolic landscape of Romanian monofloral honeys, several limitations need consideration. Firstly, only one representative sample per honey type and source was analyzed. Given the natural variability of honey composition, this restricts the statistical representativeness of the findings, and the work should therefore be regarded as a preliminary study. Moreover, all samples were harvested from Romanian regions during a single beekeeping season (2022), which introduces a degree of geographic and temporal specificity. Climatic factors such as rainfall, temperature, and soil conditions, known to influence nectar and phytochemical profiles, may further limit generalizability. The diversity of sample sources was also uneven: lavender and thyme honeys were available exclusively from local producers, reflecting their niche distribution and absence from Romanian retail chains. A more balanced representation across floral sources, seasons, and market channels would strengthen the robustness of future analyses. While the study comprehensively assessed phenolic content and antioxidant metrics, it did not include enzymatic or microbiological analyses. Enzymes like glucose oxidase and catalase, along with microbial residues, may significantly contribute to honey’s functional properties and warrant future investigation. Additionally, the antioxidant assays employed were conducted exclusively in vitro. These do not fully account for the complex dynamics of absorption, metabolism, or bioactivity in vivo. Finally, observed differences between producer and commercial samples may reflect not only botanical origin but also variations in processing, such as thermal treatment or blending. Further analytical tools, such as isotopic fingerprinting or processing markers, would help disentangle botanical effects from industrial influences.

Despite these constraints, the current work delivers a multifaceted analysis using HPLC-DAD-ESI+, three antioxidant assays, kinetic modeling, and PCA. It provides baseline evidence on the phenolic diversity and antioxidant potential of five Romanian monofloral honeys, including lesser-studied varieties such as lavender and thyme. These findings may serve as a starting point for future, larger-scale investigations aimed at substantiating the nutritional and functional relevance of Romanian monofloral honeys within the context of functional foods.

## 5. Conclusions

The present study suggests that the antioxidant activity of the selected Romanian monofloral honeys may be influenced by their botanical origin and phenolic composition. Thyme and linden honeys tended to exhibit higher phenolic levels and stronger antioxidant responses, while acacia honey showed the lowest values, with rapeseed and lavender honeys occupying an intermediate position.

The observed associations between total phenolic content, color intensity, and antioxidant performance indicate that these parameters could serve as useful indicators of honey quality. Although the sample size was limited, the integration of HPLC-DAD-ESI^+^ profiling, antioxidant assays, and multivariate analysis provides valuable initial insights into how floral source may influence the functional properties of honey. Further studies with larger and more diverse sample sets are needed to confirm and expand upon these findings.

## Figures and Tables

**Table 1:** The origin of honey samples.

Sample Code	Botanical Origin	Source
AH_P	Acacia	Producer
AH_C	Acacia	Commercial
RH_P	Rapeseed	Producer
RH_C	Rapeseed	Commercial
TLH_P	Linden	Producer
TLH_C	Linden	Commercial
LH_P	Lavender	Producer
TH_P	Thyme	Producer

**Table 2:** Reagents used in the experiments.

Molecular Formula (Reagent)/Usual Name	CAS/Concentration	Producer	Country
C_14_H_18_O_4_ (6-hydroxy-2,5,5,7,8-tetramethylchroman-2-carboxylic acid)/trolox	53188-07-1/97%	Sigma Aldrich (St. Louis, MO, USA)	USA
C_18_H_12_N_5_O_6_ (1,1-diphenyl-2-picrylhydrazyl hydrate)/DPPH	3923-52-2/99%	Sigma Aldrich	USA
C_18_H_12_N_6_ (2,4,6-tris(2-pyridyl)-S-triazine)/TPTZ	3682-35-7/98%	Sigma Aldrich	USA
C_14_H_12_N (2,9-dimethyl-1, 10-phenanthroline)/ Neocuproine	484-11-7/98%	Sigma Aldrich	USA
C_16_H_18_O (3-(3,4-dihydroxycinnamoyl) quinic acid)/ Chlorogenic acid	327-97-9/98% HPLC	Sigma Aldrich	USA
C_6_H_2_(OH)_3_COOH (3,4,5-trihydroxybenzoic acid)/ Gallic acid	149-91-7/98% HPLC	Sigma Aldrich	USA
C_15_H_10_O_6_ (3′,4′,5,7-tetrahydroxyflavone)/Luteolin	491-70-3/99% HPLC	Sigma Aldrich	USA
C_27_H_30_O1_6_ (Rutin)	153-18-4/99%	Sigma Aldrich	USA
C_18_H_18_N_4_O_6_S_4_ (2,2′-azino-bis (3-ethylbenzothiazoline-6-sulfonic acid) diammonium salt)/ABTS	30931-67-0/98%	Thermo Fisher Scientific (Waltham, MA, USA)	USA
(CH_3_)_2_SO (Dimethyl sulfoxide)/DMSO	67-68-5/99.9%	Chempur (Piekary Śląskie, Poland)	Poland
Folin–Ciocalteu reagent	12111-13-6	Carl Roth GmbH + Co KG (Karlsruhe, Germany)	Germany
FeCl_3_·6H_2_O/iron (III) chloride hexahydrate	10025-77-1/97%	Carl Roth GmbH + Co KG	Germany
CuCl_2_/copper (II) chloride	7447-39-4/97%	Carl Roth GmbH + Co KG	Germany
Na_2_CO_3_/sodium carbonate	497-19-8/99.5%	Carl Roth GmbH + Co KG	Germany
C_2_H_5_OH/Ethanol	64-17-5/70%	Chimreactiv SRL (Bucharest, Romania)	Romania
CH_3_OH/Methanol	67-56-1/90%	Chimreactiv SRL	Romania
CH_3_CN/Acetonitrile	75-05-8/99.8% HPLC	Merck (Darmstadt, Germany)	Germany
Gallic acid	149-91-7/98% HPLC	Sigma Aldrich	USA
Chlorogenic acid	327-97-9/98% HPLC	Sigma Aldrich	USA
Luteolin	491-70-3/90% HPLC	Sigma Aldrich	USA
Rutin	207671-50-9/94% HPLC	Sigma Aldrich	USA

**Table 3:** HPLC gradient program for the separation of phenolic compounds.

Time (min)	% B	Description
0	5	Initial condition
0–2	5	Isocratic
2–18	5–40	Linear gradient
18–20	40–90	Linear gradient
20–24	90	Isocratic
24–25	90–5	Linear decrease
25–30	5	Re-equilibration (isocratic)

**Table 4:** Total phenolic contents of the honey samples.

Sample	Total Phenolic Content (mg GAE/100 g)
AH_P	179.26 ± 23.57 ^a^
AH_C	234.81 ± 18.33 ^b^
RH_P	342.22 ± 13.09 ^c^
RH_C	223.70 ± 28.81 ^ab^
TLH_P	495.93 ± 31.43 ^d^
TLH_C	586.67 ± 18.33 ^e^
LH_P	329.26 ± 10.48 ^c^
TH_P	562.59 ± 20.95 ^e^

Values are expressed as mean ± SD (n = 3). Different lowercase letters (a–e) indicate statistically significant differences between samples (*p* < 0.05), based on Tukey’s multiple comparison test. Samples sharing the same letter do not differ significantly. Sample codes: AH_P—acacia honey from producer; AH_C—acacia honey from commercial source; RH_P—rapeseed honey from producer; RH_C—rapeseed honey from commercial source; TLH_P—linden honey from producer; TLH_C—linden honey from commercial source; LH_P—lavender honey from producer; TH_P—thyme honey from producer.

**Table 5:** Phenolic compounds identified in the samples by HPLC-DAD-ESI^+^.

R_t_ (min)	UV λ_max_ (nm)	[M+H]^+^ (*m*/*z*)	Compound	Subclass
3.55	270	155	2,4-dihydroxybenzoic acid	Hydroxybenzoic acid
7.41	270	171	Gallic acid	Hydroxybenzoic acid
9.55	280	155	Protocatechuic acid	Hydroxybenzoic acid
11.56	330	355	Chlorogenic acid	Hydroxycinnamic acid
12.60	270	139	*p*-hydroxybenzoic acid	Hydroxybenzoic acid
13.32	330	343	Caffeic acid-glucoside	Hydroxycinnamic acid
13.61	280	169	Vanilic acid	Hydroxybenzoic acid
14.69	340, 245	433, 271	Apigenin-glucoside	Flavone
14.90	340, 245	565, 271	Apigenin-apiosyl-glucoside	Flavone
15.23	360, 250	611, 303	Quercetin-rutinoside	Flavonol
16.02	360, 250	465, 303	Quercetin-glucoside	Flavonol
16.56	280	199	Syringic acid	Hydroxybenzoic acid
17.07	350, 250	419, 257	Pinocembrin-glucoside	Flavanone
17.27	270	139	Salicylic acid	Hydroxybenzoic acid
19.26	280	213, 199	Methyl-syringic acid	Hydroxybenzoic acid
21.86	355, 250	433, 271	Galangin-glucoside	Flavanonol
23.81	355, 250	273	Pinobanksin	Flavanonol

R_t_, retention time; λ_max_, maximum UV absorbance (nm); [M+H]^+^, protonated molecular ion (*m*/*z*); *m*/*z*—mass-to-charge ratio.

**Table 6:** Quantitative profile of total polyphenols as determined by HPLC-DAD-ESI^+^ (mg/100 g honey).

Compound	AH_P	AH_C	RH_P	RH_C	TLH_P	TLH_C	LH_P	TH_P
2,4-Dihydroxybenzoic acid	4.06 ± 0.07 ^a^	3.80 ± 0.11 ^a^	6.27 ± 0.15 ^c^	7.13 ± 0.07 ^d^	4.51 ± 0.1 ^b^	4.21 ± 0.08 ^a,b^	5.94 ± 0.23 ^c^	9.25 ± 0.36 ^e^
Gallic acid	45.90 ± 2.17 ^b^	35.78 ± 0.10 ^a^	75.53 ± 2.26 ^e^	91.64 ± 0.64 ^f^	51.31 ± 1.47 ^b,c^	54.90 ± 2.30 ^c^	66.16 ± 1.93 ^d^	127.49 ± 5.69 ^g^
Protocatechuic acid	nd	nd	nd	3.38 ± 0.1 ^d^	0.05 ± 0.00 ^a^	1.06 ± 0.02 ^b^	nd	1.89 ± 0.07 ^c^
Chlorogenic acid	nd	nd	1.17 ± 0.04 ^a,b^	4.13 ± 0.12 ^c^	26.43 ± 0.82 ^f^	23.92 ± 1.08 ^e^	6.92 ± 0.19 ^d^	1.52 ± 0.04 ^b^
*p*-Hydroxybenzoic acid	nd	nd	nd	0.42 ± 0.02 ^b^	1.15 ± 0.05 ^d^	0.67 ± 0.02 ^c^	nd	nd
Caffeic acid-glucoside	nd	nd	nd	nd	2.05 ± 0.03 ^d^	1.79 ± 0.06 ^c^	1.32 ± 0.05 ^b^	1.30 ± 0.04 ^b^
Vanilic acid	nd	nd	0.15 ± 0.01 ^b^	0.17 ± 0.00 ^b^	0.80 ± 0.01 ^d^	1.03 ± 0.04 ^e^	0.03 ± 0.00 ^a^	0.51 ± 0.01 ^c^
Apigenin-glucoside	nd	nd	0.73 ± 0.03 ^a^	3.96 ± 0.13 ^b^	29.46 ± 0.84 ^e^	21.20 ± 0.34 ^d^	8.56 ± 0.31 ^c^	0.26 ± 0.01 ^a^
Apigenin-apiosyl-glucoside	nd	nd	0.83 ± 0.02 ^b^	2.69 ± 0.08 ^c^	19.29 ± 0.41 ^e^	12.69 ± 0.46 ^d^	3.09 ± 0.11 ^c^	nd
Quercetin-rutinoside	1.07 ± 0.01 ^a^	0.89 ± 0.01 ^a^	2.82 ± 0.07 ^a^	10.02 ± 0.38 ^b^	70.5 ± 2.51 ^e^	33.93 ± 0.05 ^d^	13.16 ± 0.30 ^c^	1.26 ± 0.02 ^a^
Quercetin-glucoside	0.92 ± 0.04 ^a^	1.04 ± 0.00 ^a^	1.44 ± 0.03 ^a^	5.12 ± 0.08 ^b^	29.62 ± 1.31 ^e^	25.69 ± 0.66 ^d^	7.99 ± 0.16 ^c^	2.02 ± 0.05 ^a^
Syringic acid	nd	nd	nd	0.17 ± 0.00 ^b^	8.97 ± 0.03 ^e^	5.52 ± 0.07 ^d^	1.50 ± 0.04 ^c^	nd
Pinocembrin-glucoside	nd	nd	nd	nd	13.45 ± 0.58 ^c^	7.72 ± 0.13 ^b^	7.16 ± 0.20 ^b^	nd
Salicylic acid	4.82 ± 0.04 ^b^	8.05 ± 0.14 ^c^	9.33 ± 0.31 ^d^	22.70 ± 0.16 ^g^	12.17 ± 0.23 ^e^	12.06 ± 0.32 ^e,f^	nd	12.60 ± 0.32 ^f^
Methyl-Syringic acid	nd	nd	3.32 ± 0.04 ^b^	3.26 ± 0.1 ^b^	4.75 ± 0.1 ^c^	7.07 ± 0.31 ^d^	0.24 ± 0.01 ^a^	nd
Galangin-glucoside	2.81 ± 0.09 ^b^	nd	1.89 ± 0.06 ^a,b^	7.14 ± 0.18 ^c^	57.47 ± 1.55 ^f^	39.54 ± 1.29 ^e^	10.54 ± 0.06 ^d^	1.26 ± 0.01 ^a,b^
Pinobanksin	3.04 ± 0.09 ^a^	4.28 ± 0.04 ^b^	2.61 ± 0.08 ^a^	2.72 ± 0.1 ^a^	13.51 ± 0.32 ^d^	8.67 ± 0.35 ^c^	2.78 ± 0.1 ^a^	2.71 ± 0.09 ^a^
Total phenolic acid	54.80 ± 1.55 ^a^	47.63 ± 1.65 ^a^	95.77 ± 2.3 ^c^	132.99 ± 5.2 ^e^	112.18 ± 2.6 ^d^	112.24 ± 2.8 ^d^	82.13 ± 1.6 ^b^	154.57 ± 2.6 ^f^
Total Flavonoids	7.82 ± 0.36 ^a^	6.21 ± 0.21 ^a^	10.32 ± 0.65 ^a^	31.66 ± 0.56 ^b^	233.31 ± 2.5 ^e^	149.43 ± 1.5 ^d^	53.26 ± 1.3 ^c^	7.5 ± 0.2 ^a^
Total phenolics	62.62 ± 1.6 ^b^	53.84 ± 1.8 ^a^	106.09 ± 3.1 ^c^	164.65 ± 6.1 ^f^	345.49 ± 5.2 ^h^	261.67 ± 4.1 ^g^	135.39 ± 3.1 ^d^	162.07 ± 2.8 ^e^

Values are expressed as mean ± SD (n = 3). Different lowercase letters (a–h) indicate statistically significant differences between samples (*p* < 0.05), based on Tukey’s multiple comparison test. Samples sharing the same letter do not differ significantly. Sample codes: AH_P—acacia honey from producer; AH_C—acacia honey from commercial source; RH_P—rapeseed honey from producer; RH_C—rapeseed honey from commercial source; TLH_P—linden honey from producer; TLH_C—linden honey from commercial source; LH_P—lavender honey from producer; TH_P—thyme honey from producer, nd, not detected.

**Table 7:** Antioxidant activity of honey samples by DPPH, ABTS, and FRAP assays.

Sample	DPPH	ABTS	FRAP
	µmol TE/100 g		
AH_P	16.82 ± 0.01 ^a^	8.04 ± 0.01 ^a^	13.09 ± 0.01 ^a^
AH_C	19.60 ± 0.02 ^b^	8.30 ± 0.01 ^a^	36.09 ± 0.02 ^b^
RH_P	18.54 ± 0.01 ^a,b^	10.30 ± 0.01 ^a^	57.24 ± 0.01 ^c^
RH_C	17.14 ± 0.01 ^a^	68.34 ± 0.02 ^c^	97.55 ± 0.03 ^f^
TLH_P	20.01 ± 0.02 ^b,c^	107.03 ± 0.01 ^e^	77.07 ± 0.01 ^e^
TLH_C	19.11 ± 0.01 ^b^	91.55 ± 0.02 ^d^	67.81 ± 0.02 ^d^
LH_P	18.54 ± 0.01 ^a,b^	45.12 ± 0.01 ^b^	70.46 ± 0.01 ^d,e^
TH_P	21.82 ± 0.02 ^c^	145.72 ± 0.01 ^f^	97.55 ± 0.09 ^f^

All data are expressed as mean ± standard deviation (n = 3), in µmol Trolox equivalents per 100 g of honey. Different lowercase letters (a–f) denote statistically significant differences between samples at *p* < 0.05, as determined by Tukey’s post hoc test. Identical letters indicate no significant difference between the compared samples. AH_P—acacia honey from producer; AH_C—acacia honey from commercial source; RH_P—rapeseed honey from producer; RH_C—rapeseed honey from commercial source; TLH_P—linden honey from producer; TLH_C—linden honey from commercial source; LH_P—lavender honey from producer; TH_P—thyme honey from producer. Bold values indicate the highest activity recorded for each assay.

**Table 8:** Regression equations and parameters (*k* and *R*^2^) for the zero-, first-, and second-order kinetic models fitted to DPPH inhibition data for each honey sample.

Sample	Zero-Order Equation	*k* (Zero)	*R*^2^ (Zero)	First-Order Equation	*k* (First)	*R*^2^ (First)	Second-Order Equation	*k* (Second)	*R*^2^ (Second)
AH_P	*y* = 0.008*x* + 0.7915	0.008	0.9979	*y* = 0.0059*x* − 0.1287	0.0059	0.9932	*y* = −0.0044*x* + 1.0762	−0.0044	0.9785
AH_C	*y* = 0.0273*x* + 1.2373	0.0273	0.9985	*y* = 0.0273*x* + 1.2373	0.0273	0.9974	*y* = −0.0018*x* + 0.3908	−0.0018	0.9797
RH_C	*y* = 0.0184*x* + 1.6648	0.0184	0.9947	*y* = 0.0062*x* + 0.6226	0.0062	0.9792	*y* = −0.0022*x* + 0.5068	−0.0022	0.9527
RH_P	*y* = 0.0048*x* + 1.0462	0.0048	0.9885	*y* = 0.003*x* + 0.1159	0.003	0.9683	*y* = −0.0021*x* + 0.8729	−0.0021	0.9747
TLH_P	*y* = 0.0209*x* + 2.5655	0.0209	0.9947	*y* = 0.0052*x* + 1.0158	0.0052	0.9842	*y* = −0.0013*x* + 0.3472	−0.0013	0.9663
TLH_C	*y* = 0.0138*x* + 2.1561	0.0138	0.9993	*y* = 0.0044*x* + 0.8216	0.0044	0.9954	*y* = −0.0014*x* + 0.4255	−0.0014	0.9842
LH_P	*y* = 0.0091*x* + 1.6354	0.0091	0.9990	*y* = 0.004*x* + 0.5341	0.004	0.9934	*y* = −0.0018*x* + 0.5704	−0.0018	0.9819
TH_P	*y* = 0.0244*x* + 3.6572	0.0244	0.9964	*y* = 0.0045*x* + 1.3531	0.0045	0.9918	*y* = −0.0008*x* + 0.2497	−0.0008	0.9787

AH_P—acacia honey from producer; AH_C—acacia honey from commercial source; RH_P—rapeseed honey from producer; RH_C—rapeseed honey from commercial source; TLH_P—linden honey from producer; TLH_C—linden honey from commercial source; LH_P—lavender honey from producer; TH_P—thyme honey from producer.

**Table 9:** Antioxidant activity parameters (AUC and t_1_/_2_).

Sample	AUC (DPPH·min)	t_1/2_ (min)
AH_P	493.72	64.82
AH_C	373.36	52.99
RH_C	370.91	59.33
RH_P	287.39	126.04
TLH_P	275.11	75.74
TLH_C	211.24	92.95
LH_P	126.91	104.4
TH_P	125.27	89.48

AH_P—acacia honey from producer; AH_C—acacia honey from commercial source; RH_P—rapeseed honey from producer; RH_C—rapeseed honey from commercial source; TLH_P—linden honey from producer; TLH_C—linden honey from commercial source; LH_P—lavender honey from producer; TH_P—thyme honey from producer.

## Data Availability

The original contributions presented in this study are included in the article. Further inquiries can be directed to the corresponding authors.

## Abbreviations

The following abbreviations are used in this manuscript:

ABS_450_	Absorbance at 450 nm
ABTS	2,2′-azinobis-(3-ethylbenzthiazolin-6-sulfonic acid)
AUC	Area under the curve
DAD	Diode array detector
DPPH	2,2-Diphenyl-1-picrylhydrazyl
FRAP	Ferric reducing antioxidant power
GA	Gallic acid
GI	Gastrointestinal
HCl	Hydrochloric acid
HPLC	High performance liquid chromatography
mAU	Milli-absorbance units
MS	Mass spectrometry
Na_2_CO_3_	Sodium carbonate
NaNO_2_	Sodium nitrite
NaOH	Sodium hydroxide
PCA	Principal component analysis
SPE	Solid phase extraction
TFC	Total flavonoid content
TPC	Total phenolic content
TE	Trolox equivalents
UV-Vis	Ultraviolet-visible (spectrophotometer)
